# MiR-216b/Smad3/BCL-2 Axis Is Involved in Smoking-Mediated Drug Resistance in Non-Small Cell Lung Cancer

**DOI:** 10.3390/cancers12071879

**Published:** 2020-07-13

**Authors:** Trung Vu, Shanzhong Yang, Pran K. Datta

**Affiliations:** 1 Division of Hematology and Oncology, Department of Medicine, O’Neal Comprehensive Cancer Center, University of Alabama at Birmingham, Birmingham, AL 35294, USA; ttvu@uab.edu (T.V.); shanzhongyang@uabmc.edu (S.Y.); 2 Birmingham Veterans Affairs Medical Center, Birmingham, AL 35233, USA

**Keywords:** smoking, NSCLC, cigarette smoke condensate, miRNA, Smad3, BCL-2

## Abstract

Epidemiologic studies have shown that vast majority of lung cancers (85–90%) are causally linked to tobacco smoking. Although much information has been gained about the effects of smoking on various signaling pathways, little is known about how deregulation of miRNAs leads to activation of oncogenes and inhibition of tumor suppressor genes in non-small cell lung cancer (NSCLC). Our previous study showed that smoking inhibits TGF-β-induced tumor suppressor functions through downregulation of Smad3 in lung cancer cells. In order to understand the upstream mechanism of downregulation of Smad3 by smoking, we performed miRNA microarray analyses after treating human lung adenocarcinoma A549 and immortalized peripheral lung epithelial HPL1A cells with cigarette smoke condensate (CSC). We identified miR-216b as being upregulated in CSC treated cells. MiR-216b overexpression decreases Smad3 protein expression by binding to its 3′-UTR, and attenuates transforming growth factor beta (TGF-β) signaling and target gene expression. MiR-216b increases B-cell lymphoma 2 (BCL-2) expression and promotes chemoresistance of NSCLC cells by decreasing apoptosis. Increased acetylation of histones H3 and H4 in miR-216b gene promoter plays a role in CSC induced miR-216b expression. Taken together, these results suggest that smoking-mediated upregulation of miR-216b increases NSCLC cell growth by downregulating Smad3 and inhibiting TGF-β-induced tumor suppressor function, and induces resistance to platinum-based therapy.

## 1. Introduction

Lung cancer remains the leading cause of cancer-related deaths among both men and women in the United States, with 135,720 deaths estimated for 2020 [1]. Epidemiological studies have demonstrated that most cases of lung cancer (85–90%) are directly attributable to cigarette smoking [2,3]. Compared with non-smokers, smokers have a 10-fold greater risk of dying from lung cancer, and in heavy smokers this risk increases to 15–25-fold [3]. Although association between cigarette smoking and diseases such as lung cancer is well documented, surprisingly little is known about the mechanistic basis of smoking-related lung cancer caused by miRNAs. Cigarette smoking has been found to induce a number of genetic and molecular changes in bronchial epithelial cells, including gene mutations, promoter methylation, and chromatin modification, leading to activation of oncogenes and inhibition of tumor suppressor genes [4,5,6,7].

Members of transforming growth factor beta (TGF-β) family regulate a wide range of biological processes including cellular proliferation, cell migration, differentiation, apoptosis, and extracellular matrix deposition. Ligand binding to TGF-β receptors is required to propagate the signal to intracellular signal mediators including Smads to regulate target genes [8,9,10]. The loss of TGF-β-induced tumor suppressor functions is thought to play an important role in initiation, progression, and metastasis of lung cancer. However, little is known how TGF-β tumor suppressor functions are lost in lung cancer. Mutations in *Smad2/3* and *Smad4* genes have been found only in 5–10% of lung cancers [11,12]. Mutations within the coding sequence of TGF-β receptors (TβRI and TβRII) are very rare in non-small cell lung cancer (NSCLC) [13,14]. Deregulation of components in TGF-β signaling pathway, such as TβRII, Smad3, and Smad4, may be responsible for the loss of TGF-β-mediated tumor-suppressor functions. Our previous study showed that smoking attenuates TGF-β-induced antitumor functions through downregulation of Smad3 in lung cancer cells [15]. However, nothing is known about the mechanism by which smoking downregulates Smad3 expression in NSCLC.

MiRNAs are about 22 nucleotide long, which regulate protein expression from specific mRNA by either translational inhibition or transcript degradation. They participate in epigenetic regulation of genes and their aberrant regulation can lead to developmental abnormalities and a variety of diseases including cancer [16,17,18]. There is deregulation of miRNA expression in lung carcinoma tissues, indicating that they are involved in development and progression of lung cancer [19,20,21,22]. Several miRNAs with causal effects are upregulated (like miR-21, miR-17-92 and miR-221/222) or downregulated (like miR-34a-c, miR-29, let-7/miR-98, miR-15/16, miR128b, miR-200/429, miR-197, miR-93 and miR-126) in lung cancer [21]. Cigarette smoking can lead to deregulation of the global miRNAs in lung tissues [23]. So we hypothesize that smoking might decrease Smad3 protein expression in lung epithelial cells through upregulating miRNA expression. To test the hypothesis, we performed microarray analyses using cigarette smoke condensate (CSC) treated HPL1A and A549 cells. We observed that CSC treatment increases expression of 326 and 92 miRNAs and decreases expression of 30 and 157 miRNAs in A549 and HPL1A cells, respectively. According to TargetScan and other softwares, we found that miR-216b, which targets *Smad3* gene, is upregulated in lung cell lines treated with CSC. QRT-PCR and mutational analyses further confirms the result. Overexpression of miR-216b decreases Smad3 protein expression and inhibits TGF-β signaling in lung epithelial cells. The results show that miR-216b decreases the expression of Smad3 protein in lung epithelium, leading to inhibition of TGF-β signaling and induction in resistance to chemotherapy.

## 2. Results

### 2.1. CSC Treatment Regulates Tumor Suppressor and Oncogenic MiRNAs Identified by Microarray Analyses

**Figure 1:**
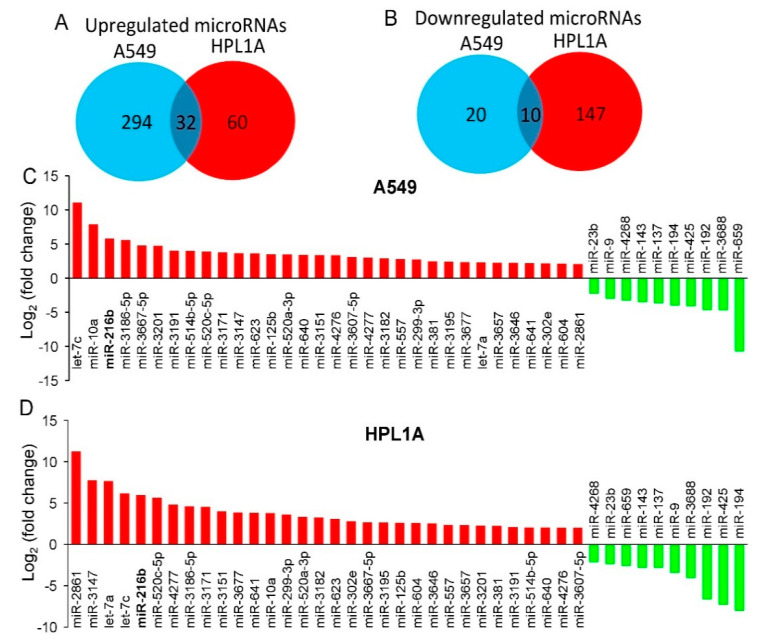
Identification of smoking-related miRNAs by microarray analyses. (**A**,**B**) Venn diagrams displaying differentially regulated microRNAs that are upregulated (log2 (fold change) > 2) (**A**) or downregulated (log2 (fold change) < −2) (**B**) in both A549 and HPL1A cells treated with CSC for 12 months. (**C**,**D**) Top common upregulated and downregulated miRNAs in CSC-treated A549 (**C**) and HPL1A (**D**) cells compared to DMSO-treated cells.

Our previous study showed that chronic treatment of lung cells with CSC decreases Smad3 expression and increases resistance to carboplatin by upregulating the expression of B-cell lymphoma 2 (BCL-2) [15]. To explore the mechanism of biological outcomes of smoking and Smad3 downregulation, we have investigated the expression of tumor suppressor and oncogenic miRNAs using microarray analyses after treating the human lung adenocarcinoma A549 and immortalized peripheral lung epithelial HPL1A cells with CSC for 12 months. These cell lines do not harbor Smad3 mutation, which allows us to analyze the effect of smoking and miR-216b on the regulation of Smad3 expression and TGF-ß signaling in both normal and cancerous lung cells. CSC treatment increases the expression of 326 and 92 miRNAs [log2 (fold change) > 2] and decreases the expression of 30 and 157 miRNAs [log2 (fold change) < −2] in A549 and HPL1A cells, respectively (Figure 1A,B and Figures S1 and S2). Based on our results, we observed that 32 microRNAs are upregulated and 10 microRNAs are downregulated in both A549 and HPL1A cell lines after CSC treatment. The relative fold changes of these common upregulated and downregulated miRNAs are shown (Figure 1C,D). We have selected these miRNAs with tumor suppressor, oncogenic, or unknown functions that are up-or down-regulated in response to CSC treatment based on the following criteria: (1) these are up-or down-regulated in both cell lines by >4-fold, (2) highest fold changes, (3) previously reported as tumor suppressor or oncomirs, and (4) those with dramatic fold changes and with unknown functions. The validity of the microarray analyses was tested by examining the expression of two downregulated miRNAs and two upregulated miRNAs (Figure S3A,B) in these two cell lines after chronic treatment of CSC by qRT-PCR assays. Out of the strongly upregulated miRNAs in both cell lines, miR-216b has a perfect binding site in the 3′UTR of Smad3, which is being characterized as follows.

### 2.2. CSC Induces MiR-216b and Reduces Smad3 Expressions in Lung Epithelial Cells

**Figure 2:**
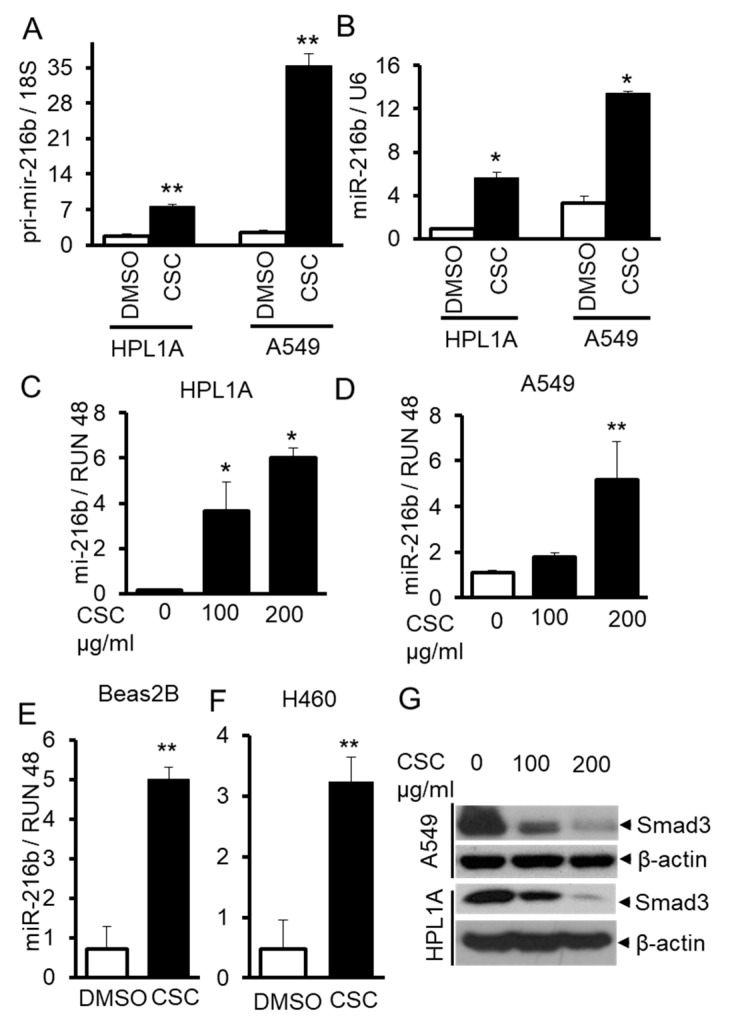
CSC induces miR-216b expression in human lung epithelial cells. (**A**,**B**) RNA was purified from HPL1A and A549 cells treated with CSC for 12 months. Pri-mir-216b and miR-216b expression levels were analyzed by real time qRT-PCR and normalized by 18S and U6, respectively (*n* = 3, the mean expression and SD are displayed). An unpaired Student’s *t*-test was used to analyze the statistical significance of differences. * *p* < 0.05, ** *p* < 0.01 *vs*. DMSO group. (**C**–**F**) HPL1A (**C**), A549 (**D**), Beas2B (**E**), and H460 (**F**) cells were exposed to different concentrations of CSC for 6 days. MiR-216b expression was measured by real time qRT-PCR and normalized by RUN 48, * *p* < 0.05, ** *p* < 0.01. Above experiments were performed at least three times with similar results. (**G**) HPL1A and A549 cells were treated with different concentrations of CSC for 6 days. Smad3 and ß-actin expression was analyzed by Western blot analysis of the cell lysates.

According to TargetScan and other softwares, we found that miR-216b, which targets *Smad3* gene, is upregulated in both cell lines treated with CSC. To confirm this result, we measured the levels of pri-mir-216b and miR-216b in long-term CSC-treated cells and control cells by qRT-PCR. As shown in Figure 2A,B, there are high levels of pri-mir-216b and miR-216b in CSC treated cells compared to those in control cells (DMSO). To further investigate whether CSC can directly induce miR-216b expression in lung epithelial cells, we treated immortalized human lung epithelium cell lines HPL1A and Beas2B and human lung cancer cell lines A549 and H460 for a short time (6 days) using high doses of CSC. MiR-216b level in the above four cell lines exposed to CSC is higher than that in control cells (Figure 2C–F). To determine the effect of CSC on Smad3 expression, we analyzed Smad3 protein levels in HPL1A and A549 cells treated with high doses of CSC for 6 days using Western blot analyses. CSC treatment decreases Smad3 expression in both cell lines when compared with control vehicle-treated cells (Figure 2G). These results suggest that CSC-induced miR-216b downregulates Smad3 expression in lung epithelial cells.

### 2.3. MiR-216b Inhibits the Expression of Smad3 in Lung Epithelial Cells

**Figure 3:**
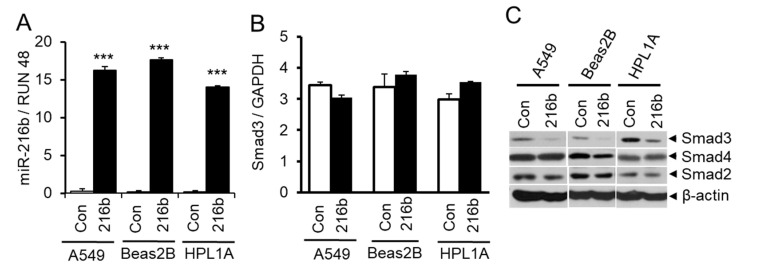
Downregulation of Smad3 by miR-216b in lung epithelial cells. (**A**) Immortalized normal human lung epithelial cell lines HPL1A and Beas2B and human lung adenocarcinoma cell line A549 were transfected with miR-216b mimic or control mimic. Forty-eight hours after transfection, RNA was purified and miR-216b expression was analyzed by qRT-PCR, *** *p* < 0.001 *vs*. control (Con) group. (**B**) Total RNA from above cell lines was used for qRT-PCR analyses of Smad3 mRNA expression and normalized by GAPDH. (**C**) HPL1A, Beas2B and A549 cells were transfected with miR-216b mimic or control mimic. Forty-eight hours after transfection, cells were harvested for protein extraction. Smad3, Smad2 and Smad4 protein levels were analyzed by Western blotting with β-actin as loading control.

To investigate whether CSC-induced miR-216b expression affects the expression of Smad3, we transfected miR-216b mimic (216b) or control miRNA mimic (Con) into A549, Beas2B, and HPL1A cells. Forty-eight hours after transfection, cells were harvested for Western blot and qRT-PCR analyses. As shown in Figure 3A, there is much higher expression of miR-216b in A549, Beas2B and HPL1A cells transfected with miR-216B mimic (216b) compared to that in the cells transfected with control miRNA mimic (Con). Interestingly, miR-216b does not affect the expression of Smad3 mRNA (Figure 3B). Next we tested the expression of Smad2, Smad3, and Smad4 in A549, Beas2B and HPL1A cells. While there is no difference in Smad2 and Smad4 protein expression by miR-216b, Smad3 expression was reduced in miR-216b-transfected cells compared to control cells (Figure 3C). These results suggest that miR-216b inhibits Smad3 protein expression through interfering Smad3 mRNA translation.

### 2.4. MiR-216b Inhibits Expression of Smad3 by Binding to Its 3′UTR

**Figure 4:**
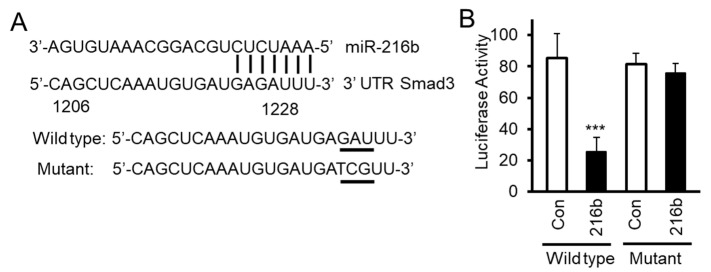
MiR-216b directly binds Smad3 gene 3′ end untranslated region (3′UTR). (**A**) We have generated luciferase constructs by inserting wild type and mutated (3 base mutation GAU**→**TCG) 3′UTR of Smad3 downstream of luciferase gene. (**B**) HPL1A cells were co-transfected with miRNA reporter vector with wild type *Smad3* gene 3′UTR containing miR-216b binding site or mutated site and miR-216b mimic or control miRNA mimic. Forty-eight hours after transfection, cells were harvested for luciferase activity assay. β-Galactosidase (β-Gal) activity was used as an internal control (*n* = 3, mean activity levels and SD are displayed). An unpaired Student’s *t*-test was used to analyze the statistical significance of differences between activity levels, *** *p* < 0.001, *vs*. control group (Con). These experiments were performed three times with similar results.

To test whether miR-216b inhibits translation of Smad3 mRNA by directly binding to its mRNA 3′UTR, we constructed luciferase reporter vector by inserting 3′UTR sequence of *Smad3* mRNA containing miR-216b binding site downstream of the luciferase gene. We also generated a mutated luciferase construct by changing three nucleotides (GAU→TCG) on Smad3 3′UTR (Figure 4A). We co-transfected these reporter vectors with Smad3 3′UTR containing wild type or mutant miR-216b binding site with miR-216b mimic (216b) or control miRNA mimic (Con) into HPL1A cells. MiR-216b inhibits luciferase activity of the reporter vector with Smad3 3′UTR containing wild type Smad3 binding site, but miR-216B has no effect on the mutant reporter (Figure 4B). These results indicate that miR-216b directly inhibits the expression of Smad3 protein through binding 3′UTR of Smad3 mRNA.

### 2.5. MiR-216b Attenuates TGF-β Signaling by Downregulating Smad3 Expression

**Figure 5:**
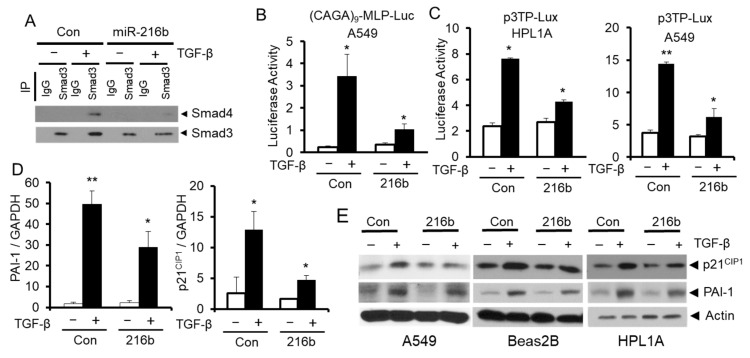
MiR-216b attenuates TGF-β/Smad signaling. (**A**) HPL1A cells were transfected by miR-216b mimic or control miRNA mimic. Forty-eight hours after transfection, cells were harvested for protein extraction. The lysates were incubated with anti-Smad3 antibody or rabbit IgG and protein-A agarose beads. The precipitated protein complexes were analyzed by Western blotting with anti-Smad4 and anti-Smad3 antibodies. (**B**,**C**) HPL1A and A549 cells were co-transfected with TGF-β signaling reporter vector containing TGF-β response element, (CAGA)_9_ MLP-Luc (**B**) or the p3TP-Lux reporter (**C**) and miR-216b mimic or control miRNA mimic. Twenty-four hours after transfection, cells were treated with 5 ng/mL TGF-β1 for 22 h. Luciferase activity was normalized to internal control β-Gal activity (*n* = 3, mean activity levels and SD are displayed), *p* < 0.05. (**D**) HPL1A cells were transfected with miR-216b mimic or control miRNA mimic. Twenty-four hours after transfection, cells were exposed to 2 ng/mL TGF-ß for 6 h and then harvested for RNA purification. p21^CIP1^ and PAI-1 mRNA levels were analyzed by qRT-PCR. (**E**) A549, Beas2B and HPL1A cells were transfected with miR-216b mimic or control miRNA mimic. Forty-eight hours after transfection, cells were exposed to 2 ng/mL TGF-β for 6 h and then harvested for Western blotting with anti-p21^CIP1^ and anti-PAI-1 antibodies.

Since miR-216b decreases Smad3 protein expression, we expect that miR-216b overexpression can inhibit TGF-β signaling in lung epithelial cells. To test this hypothesis, we first tested the effect of miR-216b overexpression on TGF-β-induced complex formation between Samd3 and Smad4 using immunoprecipitation and Western blot experiments. As shown in Figure 5A, after cells were exposed to TGF-β1, the levels of the complexes between Smad3 and Smad4 in lung epithelial cells overexpressing miR-216b were lower than that in control cells. To test whether the inhibition of Smad complex formation by miR-216b affects downstream transcriptional responses mediated by TGF-β, we conducted transient transfection assays using TGF-β-responsive reporters, p3TP-Lux and (CAGA)9-MLP-Luc. We co-transfected luciferase reporter vectors (CAGA)9-MLP-Luc or p3TP-Lux and miR-216b mimic or control miRNA mimic into HPL1A and A549 cells (Figure 5B,C). The activities of both reporters in A549 and HPL1A cell lines are reduced by miR-216b mimic when compared to those by control miRNA mimic. To determine whether miR-216b inhibits downstream TGF-β/Smad signaling target expression, we analyzed mRNA (in HPL1A cells) and protein levels of plasminogen activator inhibitor 1 (PAI-1) and p21^CIP1^ (in A549, Beas2B, and HPL1A cells) after transfecting miR-216b mimic and control miRNA mimic. We observed that miR-216b reduced the expression of PAI-1 and p21^CIP1^ mRNAs and proteins in both cell lines (Figure 5D,E). Taken together, these results suggest that miR-216b attenuates endogenous TGF-β signaling by inhibiting Smad3 expression.

### 2.6. MiR-216b Increases BCL-2 Expression and Promotes Resistance of Lung Cancer Cells to Anti-cancer Drugs by Downregulating Smad3 Expression

**Figure 6:**
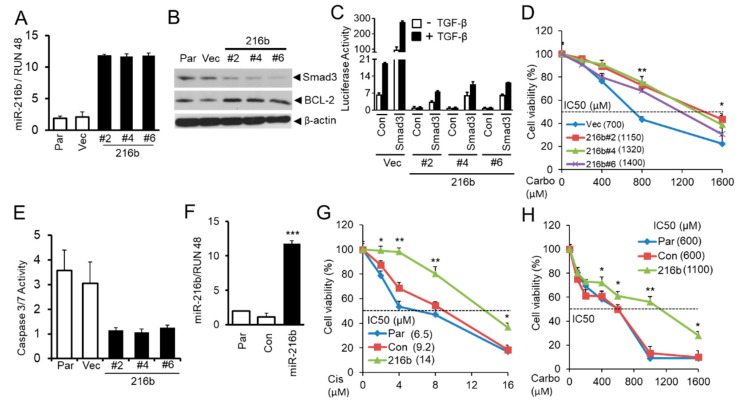
MiR-216b upregulates BCL-2 and promotes resistance of lung cancer cells to chemotherapy. (**A**) Stable expression of miR-216b in NSCLC cells H460 was tested by qRT-PCR analyses. RUN 48 was used as internal control. (**B**) Western blot analysis for Smad3 and BCL-2 protein levels in H460 cell clones stably expressing miR-216b, parental (Par), and control vector cells with β-actin as loading control. (**C**) H460 clones stably expressing miR-216b or control vector were co-transfected with TGF-β responsive luciferase reporter (CAGA)_9_ MLP-Luc and Smad3 expression vector or control vector. Twenty-four hours after transfection, cells were exposed to 5 ng/mL TGF-β1 for 22 h and then harvested for luciferase activity assay. (**D**) H460 cell clones stably expressing miR-216b or control vector were seeded into 96-well plates at 5000/well, and then cells were treated with different concentrations of carboplatin (Carbo) for 5 days. The number of viable cells was determined by MTT assay and the relative percentages of viable cells were presented. The experiment was performed three times with similar results. * *p* < 0.05, ** *p* < 0.01. (**E**) Stable clones and parental cells as described in Figure 6B were analyzed for Caspase 3/7 activity assay using Caspase-Glo 3/7 Assay kit according to the manufacturer’s instruction. (**F**) A549 cells were stably transfected with miR-216b mimic or control miRNA mimic, and the expression was tested by qRT-PCR analysis, *** *p* < 0.001. (**G**,**H**) Stably transfected A549 cells as in (**F**) above were seeded into 96-well plates and then exposed to different concentrations of cisplatin (Cis) or carboplatin (Carbo) for 3 days. MTT assays were performed for determination of the percentage of viable cells, * *P* < 0.05, ** *P* < 0.01.

To investigate whether miR-216b can increase BCL-2 expression and promote resistance of lung cancer cells to anti-cancer drugs by downregulating Smad3 expression, we established NCI-H460 human NSCLC cell clones by stably expressing miR-216b using lentiviral vector plenty-III-GFP pri-mir-216b. As shown in Figure 6A, there are high levels of miR-216b expression in the clones compared to their control counterparts. Stable expression of miR-216b in clones downregulates Smad3 level with a corresponding upregulation of BCL-2 expression (Figure 6B). To determine whether Smad3 downregulation by miR-216b results in the inhibition of TGF-β signaling, we co-transfected TGF-β signaling reporter vector (CAGA)9 MLP-Luc and Smad3 expression vector or control empty vector to vector control cells and miR-216b overexpression clones (#2, #4 and #6). After transfection, cells were exposed to TGF-β1 and normalized (with β-galactosidase) luciferase activity was determined. TGF-β-induced luciferase activity is much higher in control vector cell when compared with those in miR-216b-expressing cells (Figure 6C). Exogenous Smad3 expression restored TGF-β-induced reporter activity, suggesting miR-216b-mediated attenuation of Smad3 expression inhibits TGF-β signaling. To test whether miR-216b expression promotes chemoresistance of cancer cells, we treated H460 cell clones with miR-216b overexpression and control cells using different concentrations of carboplatin in MTT assay. Cell clones with stable expression of miR-216b show increased resistance to carboplatin (increase in IC_50_) compared to vector control cells (Vec) (Figure 6D). Concomitant with resistance induced by miR-216b, we observed that caspase 3 activity is diminished in stable clones expressing miR-216b (Figure 6E). To generalize this effect, we stably transfected A549 cells with miR-216b, and the resulting polyclonal population showed excellent expression of it (Figure 6F). We tested the effect of miR-216b on sensitivity of A549 cells to anticancer drugs. As shown in Figure 5G,H, miR-216b inhibits sensitivity of A549 cells to cisplatin and carboplatin, respectively (with increase in IC_50_). These results suggest that CSC downregulates Smad3, upregulates BCL-2, and promotes resistance of lung cancer cells to anti-cancer drugs through inducing miR-216b expression.

### 2.7. Aberrant Histone Acetylation Contributes to Elevated MiR-216b Expression in CSC Treated Lung Epithelial Cells

**Figure 7:**
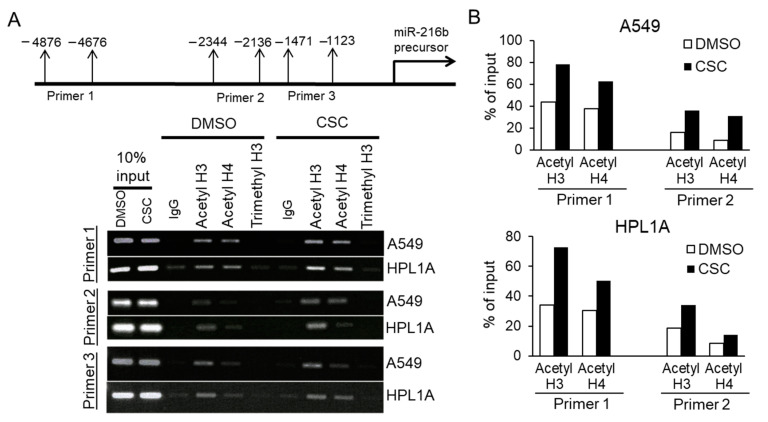
Role of histone acetylation in *miR-216b* gene expression. (**A**) Long-term CSC or DMSO-treated HPL1A and A549 cells were cross-linked by formaldehyde for 0.5 h and then terminated with glycine. ChIP assay was performed using rabbit anti-acetylated histone H3, rabbit anti-acetylated histone H4, rabbit anti-tri-methyl-histone H3 (Lys 27), and control rabbit IgG. The resulting DNA was purified and analyzed by PCR using primers (as indicated) that recognize promoter sequences of miR-216b precursor. (**B**) Densitometry of PCR products for DNA pulled down with anti-acetylated histone H3 and anti-acetylated histone H4 antibodies, and targeted by two primer sets that recognize promoter sequences of miR-216b precursor, was performed using ImageJ.

Previous studies show that cigarette smoke regulates chromatin modifications including histone acetylation/deacetylation and histone methylation/demethylation in lungs of smokers [7]. So we hypothesize that the imbalance between histone deacetylation and acetylation in favor of acetylation may contribute to CSC-induced miR-216b expression in lung cells. To test this, we analyzed the levels of chromatin acetylated Histone H3 and Histone H4 and methylated Histone H3 in the upstream promoter region of pri-mir-216b gene (distal, middle, and proximal sequences) using ChIP assay. We pulled down acetylated and methylated Histones and DNA complexes with anti-acetyl Histone H3, anti-acetyl Histone H4 or anti-H3K27me3 antibodies, and then purified DNA from above complexes. The resulting DNA was analyzed by qPCR analyses using primers that recognize different sequences of flanking upstream miR-216b promoter region (Figure 7). There is an increase in PCR products from the immunecomplex precipitated by anti-acetyl Histone H3 and H4 antibodies targeting the 5′-end upstream regions (-4876 to -4676 and -2344 to -2136) of miR-216b gene in CSC-treated A549 and HPL1A cells compared to that in DMSO-treated control cells. These results indicate that there is an increased acetylation of Histones H3 and Histone H4 on the locus of miR-216b gene in CSC treated A549 and HPL1A cells that may lead to its higher expression.

## 3. Discussion

Our previous study showed that low-dose and long-term CSC treatment downregulates Smad3 protein expression, resulting in inhibition of TGF-β signaling and decrease in TGF-β-mediated tumor suppressor functions in NSCLC [15]. In this study, we utilized the same in vitro cell culture model system to explore the mechanism by which CSC decreases Smad3 expression, increases BCL-2 expression, and enhances resistance to platinum-based therapy. Based on microarray analyses and qRT-PCR, we found that miR-216b, which targets *Smad3*, was induced by CSC. MiR-216b overexpression inhibits Smad3 protein expression in lung epithelial cells by directly binding to 3′-UTR of *Smad3* gene. Attenuation of TGF-β antitumor effects and increase in the expression of BCL-2 by miR-216b may be involved in developing resistance to cisplatin and carboplatin.

Gene expression profiles are dependent on epigenetic changes including DNA methylation, histone modification, and non-coding RNA regulation. Many studies have shown that inhibition of DNA methylations, histone deacetylation and some miRNAs can induce tumor-suppressor gene expression in lung cancer, indicating epigenetic deregulation [24,25]. Cigarette smoking has been shown to have causal effects on various cancers including lung, head and neck, esophagus, liver, pancreas, and oral cancer. Smoking is involved in epigenetic deregulation [7] that can lead to alterations in global miRNA expression in airways and lung tissues [24,26,27,28]. In this study, we used our cell culture model system to investigate changes in miRNA expression profile in normal lung epithelial cells (HPL1A) and lung adenocarcinoma cells (A549) exposed to low dose of CSC for 12 months to mimic the conditions of long-term cigarette smoking [15]. According to miRNA microarray analyses, 92 miRNAs are upregulated and 157 miRNAs downregulated in CSC-treated HPL1A cells, whereas in A549 cells, 326 miRNAs are upregulated and 30 miRNAs are downregulated (Figure 1A,B and Figures S1 and S2). The miRNA profiles from these two cell lines are substantially different, probably because one is immortalized lung epithelial cell line and the other is adenocarcinoma cell line, and their genetic backgrounds are different. Based on our result, 32 miRNAs are upregulated and 10 miRNAs are downregulated in both A549 and HPL1A cell lines after long-term CSC treatment (Figure 1). Importantly, among the upregulated miRNAs, miRNA-1180, miR-342-5p, and miRNA-1285-3p (highlighted in Figure S1) were previously shown to be associated with smoking status and reduced pulmonary functions [29]. In addition, among the downregulated miRNAs in our system, miR-362-3p, miR-150, miR-15a, and miR-10b were reported to be downregulated in bronchial airway epithelium of current smokers compared to never smokers [30]. These observations indicate that this cell culture model can be used to represent the effect of long-term smoking on the regulation of microRNA expression, leading to downstream oncogenic signaling. According to TargetScan and other softwares, we found that miR-216b, which is strongly upregulated in both cell lines in response to CSC (HPL1A: 62 fold and A549: 56 fold), has a predicted binding site in the 3′UTR of *Smad3* gene. To confirm and generalize this result, we measured the levels of primary and mature miR-216b in CSC-treated four lung cell lines by qRT-PCR analyses (Figure 2). It has been shown that increased acetylation of histones H3 and H4 and a decrease in histone deacetylase activity have been directly correlated with regulation of proinflammatory gene expression in lungs in vivo and in lung cells in vitro during cigarette smoke exposure [31,32]. Here, we show that there are increased levels of acetylated histones H3 and H4 in miR-216b gene promoter (Figure 7), indicating CSC induces miR-216b expression through remodeling chromatin in miR-216b gene promoter.

The resistance to TGF-β-mediated tumor suppressor function could be caused in multiple ways involving both genetic and epigenetic changes in TGF-β signaling molecules. However, mutation and deletion with the coding sequence of the main components of TGF-β signaling pathway including TβRII, Smad2/3 and Smad4 are rare or less often in NSCLC. Recent studies show that interaction between TGF-β signaling and miRNAs is involved in the development of diseases, such as cardiovascular diseases, pulmonary diseases and cancer [33,34,35]. Several miRNAs have been identified to decrease the expression of the main components of TGF-β signaling pathway, such as members of the miR-17-92 family [36]. These miRNAs overexpress in tumors and play critical roles in the loss of TGF-β-mediated tumor suppressor functions [37,38]. Our current study shows that the overexpression of miR-216b significantly decreases the protein levels of Smad3 (Figure 3C and Figure 6B), whereas the Smad3 mRNA level remains unchanged (Figure 3B) in four different cell lines, demonstrating that miR-216b targets Smad3 by inhibiting its translation and not by degrading its mRNA. This observation is consistent with other studies in which miR-216b was found to suppress the translation of its target genes such as *Kras* [39] and *Igf2bp2* [40] without affecting the mRNA levels, despite the perfect base pairing between the seed sequence of mature miR-216b and the 3′UTR of target mRNAs predicted by computational algorithms. Based on the current understanding of miRNA-mediated regulation of gene repression, it is essentially unknown what exactly determines whether a targeted mRNA will be translationally repressed or directed to mRNA degradation [41]. In vitro studies showed that the degree of complementarity of the seed region is a structural key determinant in selecting the route for gene suppression [41]. Therefore, it is possible that the binding between mir-216b and 3′UTR of these target mRNAs is not strong enough for RISC to cause mRNA degradation at the target site [42].

Our previous study showed that loss of Smad3 in cigarette smoke condensate (CSC)-treated cells increases the expression of BCL-2. However, this link between *Smad3* and *BCL-2* mRNA levels in lung tumors is just an overall inverse correlation [15], which might be the results of miR-216b-independent functions mediated by smoking. In addition, the mRNA levels of both *Smad3* and *BCL-2* could be regulated by other factors including genetic background, epigenetic environment, and differential regulation of oncogenes and tumor suppressor genes. Moreover, a number of oncomirs and tumor-suppressor miRNAs are differentially regulated by CSC (microarray analyses, Figure 1 and Figures S1 and S2), which could contribute to Smad3 and BCL-2 regulations. Taken together, our data shows that upregulation of miR-216b by CSC suppresses Smad3 protein level and upregulates BCL-2 level in NSCLC cell lines.

We next corroborate the direct binding between miR-216b to 3′UTR of *Smad3* by using mutated sequences in the luciferase assays (Figure 4). The specificity of this effect was reinforced by the observation that overexpression of miR-216b in three lung cell lines downregulates Smad3, and not Smad2 or Smad4 (Figure 3). As a result, we have observed decreased Smad3/Smad4 complex formation in response to TGF-β and reduced TGF-β-induced transcription from p3TP-Lux and (CAGA)9-MLP-Luc reporters (Figure 5A–C). The inhibitory effects of miR-216b on TGF-β signaling and its tumor-suppressor functions is confirmed by the fact that the expression of endogenous PAI-1 and p21^CIP1^ in response to TGF-β is decreased significantly by miR-216b in lung cell lines (Figure 5D,E). These results indicate that CSC-induced miR-216b directly inhibits the expression of Smad3 protein through binding to its mRNA.

Our previous study showed that CSC can induce anti-apoptotic gene *BCL-2* expression and promote lung cancer cell chemoresistance through downregulating Smad3 expression [15]. Upregulation of BCL-2 has been found to be involved in the mediation of chemotherapy resistance in human lung cancer [43] and considered as a target for overcoming chemoresistance in lung cancer [44]. Since CSC treatment downregulates Smad3 by promoting miR-216b expression, we reasoned that induction in miR-216b function might upregulate BCL-2 by reducing Smad3 expression and increase chemoresistance of lung cancer cells. To test this hypothesis, we established stable clones with miR-216b overexpression in lung cancer cell line H460. The result shows miR-216b introduction downregulates Smad3, increases BCL-2 level, induces chemoresistance to carboplatin with an increase in IC_50_, and decreases apoptosis (Figure 6A–E), indicating that miR-216b is a potential upstream regulator of BCL-2 in lung cancer. In an attempt to generalize this observation, we observed that overexpression of miR-216b in A549 cells increases resistance to cisplatin and carboplatin (Figure 6F–H). To our knowledge, this is the first time miR-216b expression is correlated with TGF-β/Smad3 signaling in lung cell lines. These results further suggest that miR-216b upregulation may in part contribute to lung cancer chemoresistance induced by cigarette smoking, and targeting miR-216b can sensitize lung cancer cells to platinum-based chemotherapy. Therefore, mouse experiments using xenografts derived from lung cancer cells are required in the future to validate the effect of miR-216b overexpression on the sensitivity of lung cancer cells to chemotherapy in vivo.

MiR-216b has been reported to be regulated in several cancer types including NSCLC. Liu et al. recently showed for the first time the clinical results from serum exosomal miR-216b in NSCLC [45]. The study showed that compared to NSCLC patients at the advanced stages (III/IV), serum exosomal miR-216b levels are higher in NSCLC patients at the early stages (I/II). These results will increase our understanding of how cigarette smoke causes deregulation of miRNAs in lung cells by remodeling chromatin, leading to inhibition of TGF-β signaling and loss of TGF-β mediated tumor suppressor functions. Our results suggest that miR-216b can be used as a biomarker for treating patients with NSCLC. These studies suggest that patients with lung cancer, who are smokers and have high expression of miR-216b and BCL-2, would be resistant to platinum-based chemotherapy. The therapeutic efficacy of these patients could be improved by BCL-2 inhibitor. In addition, there is a vital need for the development of a sensitivity test to identify NSCLC patients who are at risk of initiating metastasis. The miRNA signature obtained by microarray can also be verified in further studies using patient lung tumor tissue or blood for selecting a group of patients for personalized treatment.

## 4. Materials and Methods

### 4.1. Cell Lines and Tissues

Human lung cancer cell lines A549, NCI-H460, and immortalized human lung epithelial cell lines Beas2B and HPL1A were maintained in RPMI 1640 with 10% FBS. To establish stable cell lines with miR-216b overexpression, NCI-H460 cells were infected with lenti-virus expressing miR-216b or GFP lenti-virus. Forty-eight hours after infection, cells were selected using 1.5 µg/mL puromycin for 14 days. Positive clones were tested and maintained in RPMI 1640 medium with 0.5 µg/mL puromycin.

### 4.2. Reagents and Antibodies

TGF-β1 was purchased from R&D Systems (Minneapolis, MN). Human has-miR-216b mimic and control miRNA mimic were from QIAGEN. Antibodies were purchased as follows: anti-Smad2 (Cat# L16D3) and anti-Smad3 (Cat# 9513S) from Cell Signaling; anti-p21^CIP1^ (Cat# sc-6246) and anti-Smad4 (Cat# sc-7966) from Santa Cruz Biotechnology; and anti-β-actin (Cat# A1978) from Sigma Biochemicals. Cigarette smoke condensate (CSC) was purchased from Murty Pharmaceuticals, Inc.

### 4.3. Immunoblot Analysis

For immunoblotting, whole-cell lysates were prepared in cold lysis buffer supplemented with protease inhibitors (Aprotinin, Leupeptin, and PMSF) by sonication as we described [46]. The proteins were separated by 10% sodium dodecyl sulfate-polyacrylamide gel electrophoresis and transferred to PVDF membrane (Bio-Rad, Hercules, CA, USA). Membranes were then blocked with 5% nonfat dry milk in PBST, followed by blotting with primary antibodies overnight at 4 °C. After washing, membranes were incubated with species-specific secondary antibodies for 1 h at room temperature. The signals were visualized by enhanced chemiluminescence assays. The whole blot can be found at Supplementary Materials (see Figures S4–S7).

### 4.4. Immunoprecipitation

For immunoprecipitation experiments, A549 cells were transfected with miR-216b mimic or control miRNA mimic using HiperFect^®^ Transfection Reagent (QIAGEN). The cells were treated with 5 ng/mL TGF-β1 for 1 h before harvesting. Cells were lysed in 0.5 mL of extraction buffer. The lysates were incubated on ice for 30 min and subsequently cleared by centrifugation at 12,000 rpm for 15 min at 4 °C. Ten microliters of protein A agarose beads (MILLIPORE) were added to 500 μL of cellular lysate (1 mg of protein) and rotated at 4 °C for 2 h. The samples were centrifuged for 1 min at 1500 rpm. Primary anti-Smad3 antibody or rabbit IgG was added to the supernatant, and the mixture was rotated overnight at 4 °C. Ten microliters of protein A agarose beads were added and rotated at 4 °C for 1 h. The beads were pelleted by gentle centrifugation and washed three times with 1 mL of ice-cold extraction buffer. After the final wash, the precipitated protein complexes were resuspended in SDS sample loading buffer, boiled for 5 min, and loaded on to SDS-PAGE gel for Western blotting.

### 4.5. Plasmids

Human pri-mir-216b cDNA was amplified by PCR and then inserted into pLenti-III-mir cloning vector through EcoRI and Xhol restriction enzyme sites, leading to the establishment of has-miR-216b lentivector. MiRNA luciferase reporter vector with *Smad3* gene 3′ untranslated region (3′UTR) containing miR-216b binding site was constructed by inserting it downstream of luciferase gene. *Smad3* gene 3′UTR containing a mutated site was generated using two-step PCR-based site-directed mutagenesis method and then inserted into luciferase vector.

### 4.6. Quantitative Real-time PCR Analysis

Taqman probes for RUN 48 and miR-216b were purchased from Applied Biosystems (Foster City, CA, USA). mRNA levels of *Smad3*, *p21^CIP1^*, *PAI-1*, and *GAPDH* were determined by real-time PCR using SYBR Green master mix kit (Roche, Indianapolis, IN). Primer sequences were as follows: human *p21^CIP1^*: sense, 5′-GAC ACC ACT GGA GGG TGA CT-3′, antisense, 5′-CAG GTC CAC ATG GTC TTC CT-3′; human *Smad3*: sense, 5′-G AAC AGC TGT GTC TGC CAA A-3′, antisense, 5′-TGG ACT GTG ACA TCC CAG AA-3′; human *PAI-1*: sense, 5′-GAC ATC CTG GAA CTG CCC TA-3′, antisense, 5′-GGT CAT GTT GCC TTT CCA GT-3; and human *GAPDH*: sense, 5′-CGA GAT CCC TCC AAA ATC AA-3′, antisense, 5′-TGT GGT CAT GAG TCC TTC CA-3′.

### 4.7. Luciferase Reporter Assay

Cells were transiently transfected with various constructs and β-galactosidase (β-Gal) vector using Lipofectamin 2000 (Invitrogen). In each experiment, equal amounts of total DNA were transfected. Forty-eight hours after transfection, cells were harvested for luciferase activity assay. Luciferase activity was normalized to β-gal activity and the relative luciferase activity was presented as the average of triplicate values.

### 4.8. Chromatin Immunoprecipitation (ChIP) Assay

ChIP was performed as described in previous study [47]. In brief, long-term CSC or DMSO-treated HPL1A and A549 cells were cross-linked by 1% formaldehyde on ice for 30 min. Cross-linking was terminated by glycine (final concentration 0.125 M). After washed three times with PBS, cells were scraped into buffer 1 containing protease inhibitors and rocked at 4 °C for 10 min. Cells were collected by centrifugation and re-suspended in buffer 2 containing protease inhibitors. Nuclei were collected by centrifugation, re-suspended in buffer 3 containing protease inhibitors, and sonicated on ice to an average length of 1000 bp. After centrifugation, supernatant was adjusted to RIPA buffer. After being pre-cleared with protein agarose beads (blocked previously with 1 mg/mL salmon sperm DNA and 1 mg/mL BSA) at 4 °C for 3 h, samples were incubated with 2 μg/mL of each antibody including anti-acetyl Histone H3, anti-acetyl Histone H4, anti-Tri-methyl-Histone H3 (Lys 27), and control IgG at 4 °C overnight. Next day, 20 μL protein A agarose beads were added, and immune complexes were recovered, washed under stringent condition, treated with proteinase K at 55 °C for more than 4 h, and followed by overnight reversal of cross-links. The resulting DNA was purified and analyzed by PCR using primers that recognize promoter sequences of miR-216b precursor.

### 4.9. Statistical Analysis

Results were statistically compared using an unpaired Student’s *t*-test or One-Way ANOVA. A *p*-value of *p* < 0.05 was considered significant. All data are representative of at least three independent experiments and are expressed as the mean ± SD unless otherwise mentioned.

## 5. Conclusions

The novelty of this study is to identify miR-216b as an upstream regulator of Smad3 and BCL-2 expressions in response to cigarette smoking, which contributes to the inhibition of TGF-ß signaling and resistance to chemotherapy. This study provides a hint of how smoking may regulate the level of miR-216b epigenetically through acetylation of histones H3 and H4. Moreover, this investigation reveals a miRNA profile in NSCLC with tumor-suppressor and oncogenic functions potentially regulated by smoking, which will be informative for future studies. Altogether, these results suggest a new mechanism of action by which cigarette smoking regulates a tumor-suppressor pathway by modulating miRNA. The knowledge gained from this study can be applied for using miR-216b and BCL-2 as markers for detecting patients who might have resistance to platinum-based chemotherapy and improving the therapeutic efficacy in patients by targeting those players.

## Supplementary Materials

The following are available online at https://www.mdpi.com/2072-6694/12/7/1879/s1, Figures S1 and S2: Heatmaps depicting expression changes of differentially expressed miRNAs (log2 (fold change) > 2 or log2 (fold change) < −2) in A549 (S1) and HPL1A (S2) cells treated with CSC for 12 months compared to DMSO treated cells. Figure S3: CSC regulates expression of different miRNAs in human lung epithelial cells. Figure S4–S7: Western blot files with densitometry data.
