# Supplementary material for: MiR-216b/Smad3/BCL-2 Axis Is Involved in Smoking-Mediated Drug Resistance in Non-Small Cell Lung Cancer
Source: Cancers (Basel). 2020 Jul 13;12(7):1879. doi: 10.3390/cancers12071879 (PMC7408725; doi:10.3390/cancers12071879)
Supplement: Supplementary file 1 [file cancers-12-01879-s001.pdf]

cancers

MDPI

*Article*

# MiR-216b/Smad3/BCL-2 Axis is Involved in Smoking-mediated Drug resistance in Non-Small Cell Lung Cancer

**Trung Vu, Shanzhong Yang and Pran K. Datta**

**Supplementary Materials:**

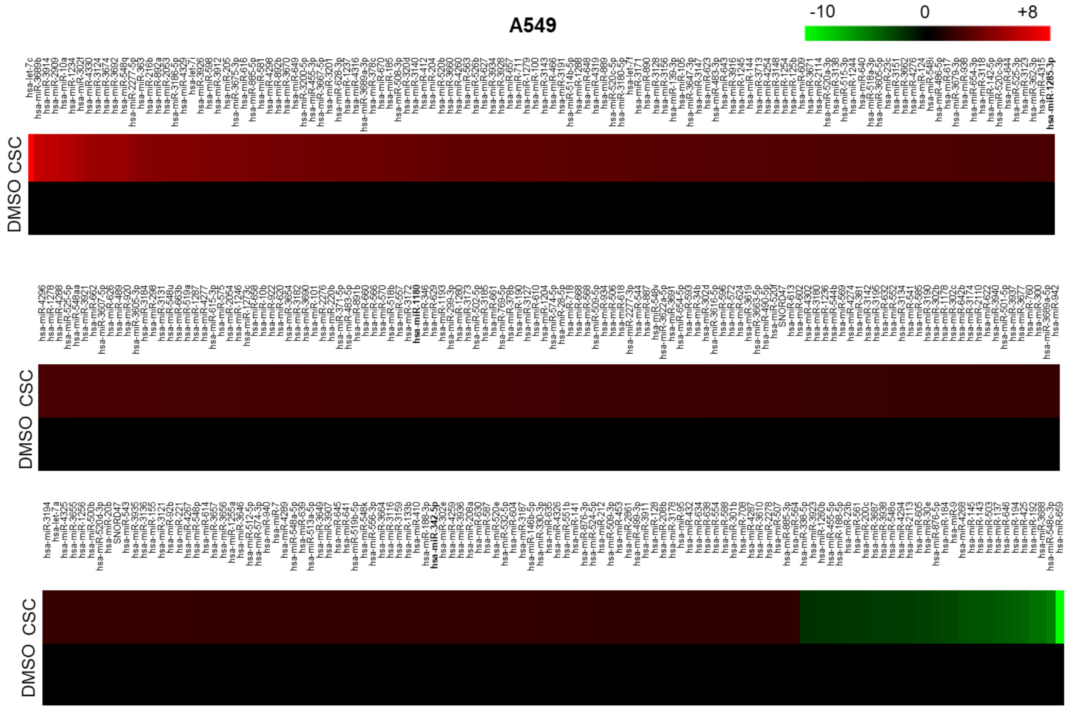


**Figure S1.** Differential upregulation of miRNAs in response to smoking. Heatmaps depicting expression changes of differentially expressed miRNAs (log2 (fold change) > 2 or log2 (fold change) < -2) in A549 cells treated with CSC for 12 months compared to DMSO treated cells. The miRNAs that were previously shown to be associated with smoking status in previous reports were highlighted.

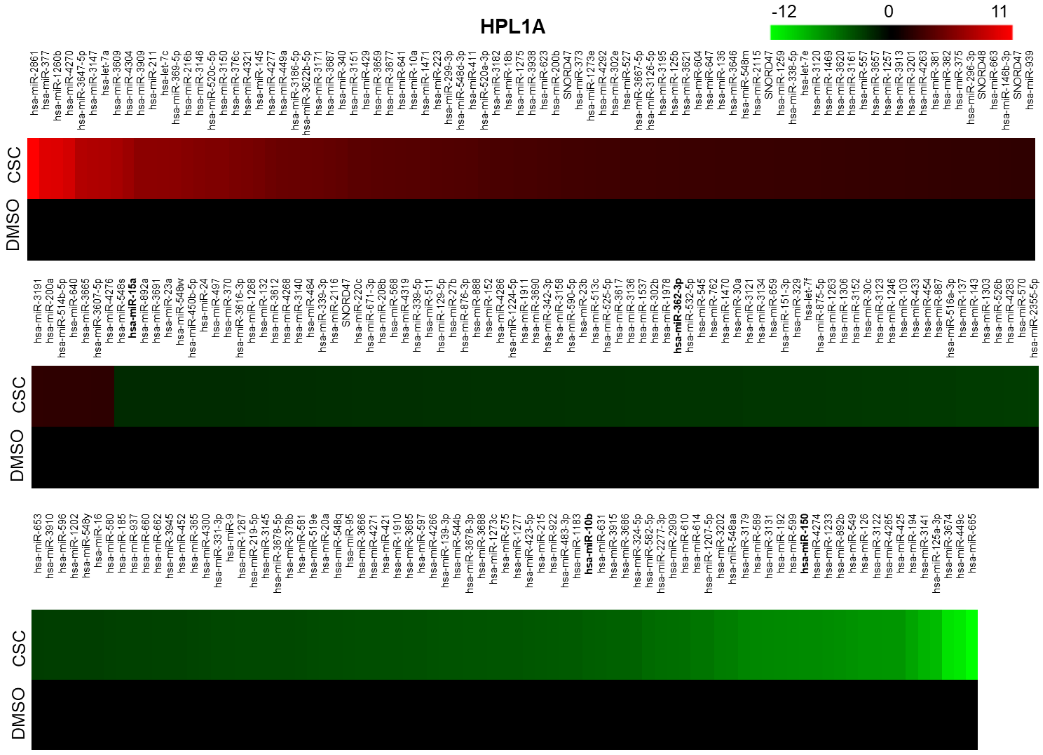


**Figure S2.** Differential downregulation of miRNAs in response to smoking. Heatmaps depicting expression changes of differentially expressed miRNAs (log2 (fold change) > 2 or log2 (fold change) < -2) in HPL1A cells treated with CSC for 12 months compared to DMSO treated cells. The miRNAs that were previously shown to be associated with smoking status in previous reports were highlighted.

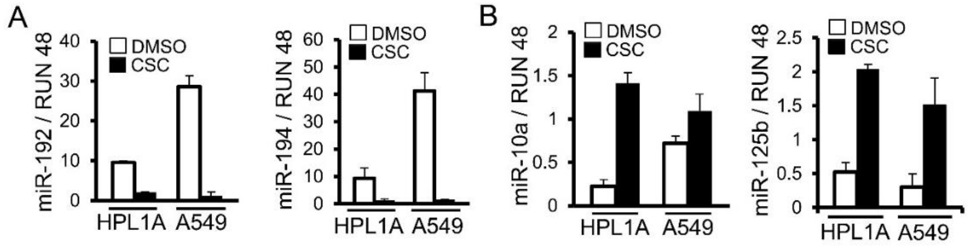


**Figure S3.** Verification of the expression of two up- and down-regulated miRNAs obtained from microarray data. CSC regulates expression of different miRNAs in human lung epithelial cells. qRT-PCR analysis for miR-192, miR-194, miR-10a, and miR-125b expression in A549 and HPL1A cells treated with CSC or DMSO. RUN 48 was used as internal control.

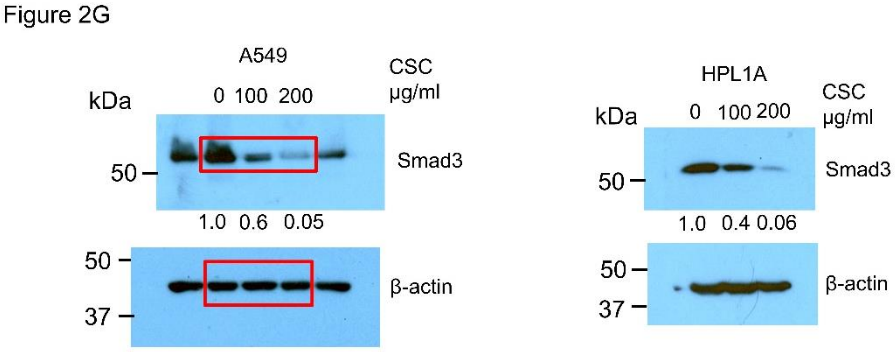


**Figure S4.** Original western blots for Figure 2G showing all bands.

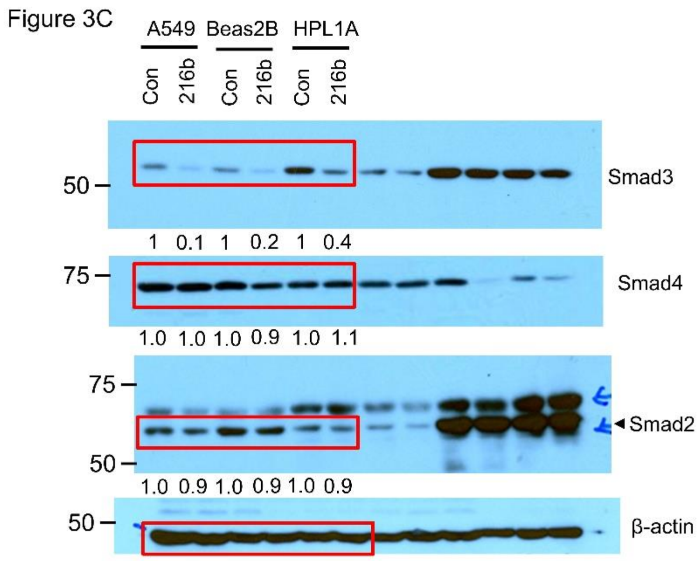


**Figure S5.** Original western blots for Figure 3C showing all bands.

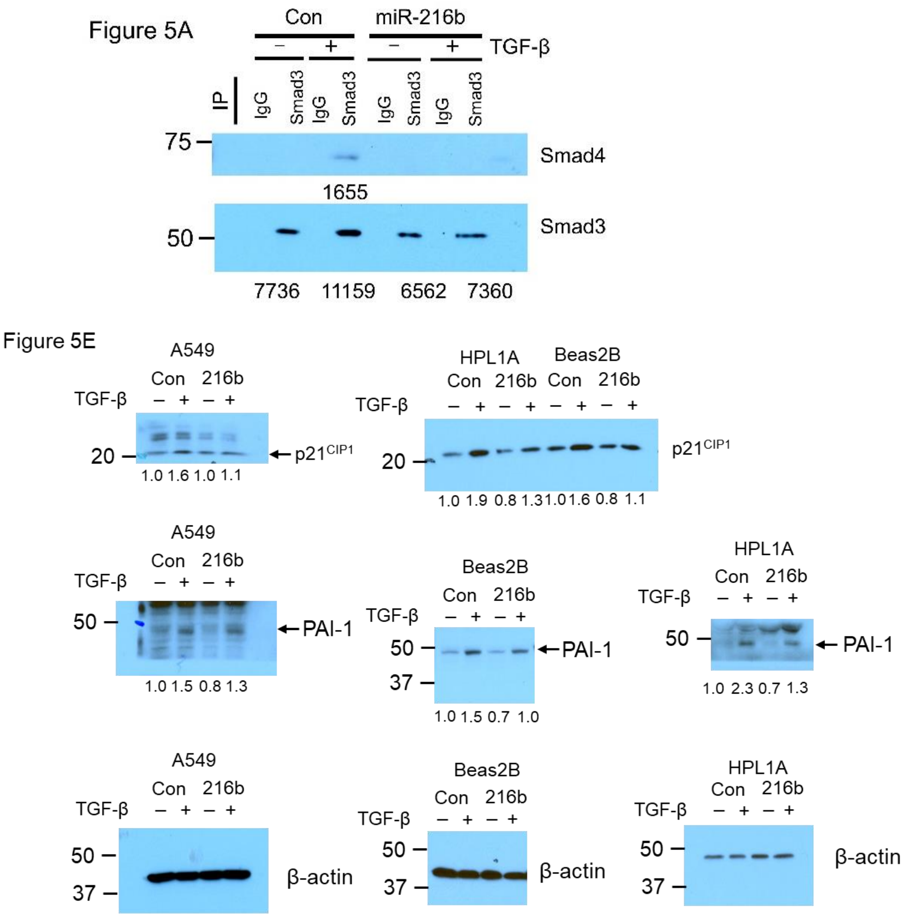


**Figure S6.** Original western blots for Figure 5 showing all bands.

Figure 6B

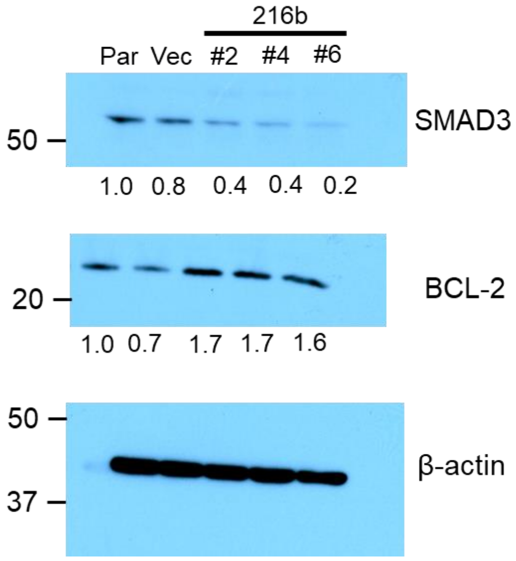


**Figure S7.** Original western blots for Figure 6B showing all bands.

© 2020 by the authors. Submitted for possible open access publication under the terms and conditions of the Creative Commons Attribution (CC BY) license (http://creativecommons.org/licenses/by/4.0/).
